# RETRACTION: Epinephrine Enhances the Response of Macrophages under LPS Stimulation

**DOI:** 10.1155/bmri/9864769

**Published:** 2026-06-12

**Authors:** BioMed Research International

RETRACTION: J. Zhou, J. Yan, H. Liang, and J. Jiang, “Epinephrine Enhances the Response of Macrophages under LPS Stimulation,” *BioMed Research International* 2014, no. 1 (2014): 1‐8, 10.1155/2014/254686

The above article, published online on 26 August 2014 in Wiley Online Library (wileyonlinelibrary.com), has been retracted by agreement between the journal Editor, Yoel A. Klug and John Wiley & Sons Ltd.

The retraction has been agreed upon following an investigation into image inconsistencies. Figures 1a, 1e and 5b show unexpected similarities between macrophages within the same images.

The authors responded to state that the images were not artificially modified and that similarity likely stems from the inherent morphological resemblance of primary peritoneal macrophages. This response was not consistent with the results of our investigation, and the article is therefore retracted.

The authors disagree with the retraction.

